# Population of Computational Rabbit-Specific Ventricular Action Potential Models for Investigating Sources of Variability in Cellular Repolarisation

**DOI:** 10.1371/journal.pone.0090112

**Published:** 2014-02-28

**Authors:** Philip Gemmell, Kevin Burrage, Blanca Rodriguez, T. Alexander Quinn

**Affiliations:** 1 Department of Computer Science, University of Oxford, Oxford, United Kingdom; 2 School of Mathematical Sciences, Queensland University of Technology, Brisbane, Australia; 3 Department of Physiology and Biophysics, Dalhousie University, Halifax, NS, Canada; Georgia State University, United States of America

## Abstract

Variability is observed at all levels of cardiac electrophysiology. Yet, the underlying causes and importance of this variability are generally unknown, and difficult to investigate with current experimental techniques. The aim of the present study was to generate populations of computational ventricular action potential models that reproduce experimentally observed intercellular variability of repolarisation (represented by action potential duration) and to identify its potential causes. A systematic exploration of the effects of simultaneously varying the magnitude of six transmembrane current conductances (transient outward, rapid and slow delayed rectifier K^+^, inward rectifying K^+^, L-type Ca^2+^, and Na^+^/K^+^ pump currents) in two rabbit-specific ventricular action potential models (Shannon *et al.* and Mahajan *et al.*) at multiple cycle lengths (400, 600, 1,000 ms) was performed. This was accomplished with distributed computing software specialised for multi-dimensional parameter sweeps and grid execution. An initial population of 15,625 parameter sets was generated for both models at each cycle length. Action potential durations of these populations were compared to experimentally derived ranges for rabbit ventricular myocytes. 1,352 parameter sets for the Shannon model and 779 parameter sets for the Mahajan model yielded action potential duration within the experimental range, demonstrating that a wide array of ionic conductance values can be used to simulate a physiological rabbit ventricular action potential. Furthermore, by using clutter-based dimension reordering, a technique that allows visualisation of multi-dimensional spaces in two dimensions, the interaction of current conductances and their relative importance to the ventricular action potential at different cycle lengths were revealed. Overall, this work represents an important step towards a better understanding of the role that variability in current conductances may play in experimentally observed intercellular variability of rabbit ventricular action potential repolarisation.

## Introduction

Variability is perhaps an essential component of physiological systems. It is observed at all levels of spatial and temporal organisation, from sub-cellular processes to the whole-organism, and over time scales spanning from nanoseconds to years. In most cases, however, the underlying causes of physiological variability remain unclear. Moreover, its importance in health and disease, where it may explain the spectrum of responses often seen between individuals, is largely unknown. Thus, it has long been ignored in experimental and computational research.

The method usually taken by experimentalists in dealing with variability involves averaging across many subjects, thus determining the mean response. Subsequently, computational models are generated based on reported mean values, creating representations of the ‘typical’ case that fail to account for underlying variability [1]. This approach results not only in a loss of information, but in an inability of models to explain physiological observations that may depend on the presence of variability. This is certainly the case in the field of computational cardiac electrophysiology modelling. At all levels of integration, variability in cardiac activity exists, whether it is across the heart, between individual cells, or within ion-channels.

Even so, by extending investigations beyond that which is experimentally feasible, computational research is becoming an increasingly valuable tool for improving our understanding of cardiac electrophysiology [2]. While it may be that current cell models are limited in that they generally produce only an ‘average’ action potential (AP), efforts are underway to improve their scope by representing variability in cellular processes. This is being approached in multiple ways: i) by the inclusion of stochasticity in model formulations [3]–[9]; ii) by the use of parameter sensitivity analyses [10]–[14]; and iii) by the generation of model populations representing observed variability [4], [15]–[19]. The most extensive example to date of the use of a model population to investigate cardiac electrophysiological variability comes from a study by Britton *et al.* [15], in which a large population of rabbit-specific Purkinje AP models was generated by randomly assigning specific parameter values to various ionic current conductances and channel kinetics. This was followed by constraint of the model population using experimental data (a validation step essential for model development [1], [2]). Results demonstrated that particular combinations of parameters (‘parameter sets’) appeared to determine AP shape and rate-dependence, and that under conditions of K^+^ channel block the model population was able to predict experimentally measured AP prolongation.

The current study is focused on understanding variability of ventricular AP repolarisation, as increased variability in the ventricles has been related to increased arrhythmic risk [20]–[31]. Our specific aim was to generate physiologically relevant populations of computational rabbit-specific ventricular AP models with variable transmembrane current conductances that reproduce experimentally observed intercellular variability of repolarisation (represented by AP duration, APD) to explore its potential sources. We present a framework for systematic parameter space exploration using distributed computing software [32]–[34] and specialised visualisation techniques [35]–[38], specifically designed for large-scale parameter sweeps and grid execution, along with model calibration using experimental data. This allowed investigation of the interactions between the varied conductances, their relative importance to AP repolarisation, and rate- and model-dependent effects.

## Materials and Methods

### Exploring Ventricular AP Response to Simultaneous Variations in the Magnitude of Transmembrane Current Conductances Important for Repolarisation

Two biophysically-detailed computational cell models were used in this study to simulate the AP of a rabbit ventricular epicardial myocyte (allowing assessment of model-dependent effects). The first was created by Shannon *et al.* [39] and the second was an updated version of that model by Mahajan *et al.* [40], which includes updates to the L-type Ca^2+^ current, intracellular Ca^2+^ cycling, Na^+^- Ca^2+^ exchanger, and channel distributions updated to better replicate AP and Ca^2+^-handling dynamics at rapid stimulation rates. Importantly, of the small animals, rabbit has cardiac electrophysiology most similar to human, and thus is a preferred model for experimental research and pharmacological testing [41], providing established reference values for constraining models to a physiological range.

Simulations were designed to examine the response of these models to simultaneous variation of the magnitude of multiple transmembrane current conductances important for ventricular repolarisation. The currents considered (with their conductance given in parentheses) were: the transient outward current (*g*_to_); the rapid delayed rectifier K^+^ current (*g*_Kr_); the slow delayed rectifier K^+^ current (*g*_Ks_); the inward rectifying K^+^ current (*g*_K1_); the L-type Ca^2+^ current (*g*_Ca,L_); and the Na^+^/K^+^ pump current (*g*_NaK_). Synthesising the information available in the literature to create well-defined ranges is difficult because available experimental values for these current conductances come from various laboratories and are often produced using vastly different (and sometimes ill-reported) methods and conditions ([42]). Thus, conductances were varied by 0%, ±15%, and ±30%, which is within the bounds of experimentally reported variability in rabbit ventricular myocytes [43], aligns with previous computational investigations [11], and provided a good compromise between the size of the parameter space and computational tractability. Current formulations, on the other hand, were left unchanged, based on the assumption that AP variability is primarily a result of differences in the relative magnitude of currents, rather than underlying current dynamics. This generated a population of 15,625 models for both the Shannon and Mahajan formulations. Model APD was compared to experimentally reported values to define physiological parameter sets, and in cases where no parameter sets generated matches, the range of conductance variation was expanded until matches were found (described further in the next section). Simulations were performed at a cycle length (CL) of 400, 600, and 1,000 ms to constrain the populations of models and to examine potential rate-dependent effects.

Both models were downloaded from the CellML model repository (http://models.cellml.org/cellml). [K^+^]_i_ was unclamped and the Shannon model was corrected as suggested previously [44]. The CellML files were converted to C++ using the Cellular Open Resource (COR) software (http://cor.physiol.ox.ac.uk/) [45] and simulations were performed using an ordinary differential equation solver with adaptive time-stepping (Sundials CVODE, version 2.4.0) and relative and absolute tolerances set to 10^−7^ and 10^−9^, respectively. Simulation duration was set to 1,000 s and run across all parameter sets using the Nimrod/G distributed computing grid [32], [34], part of a suite of software tools developed by the Monash eScience and Grid Engineering Laboratory for parameter sweeps and grid execution, including scheduling across multiple computer resources [33]. Simulated APs with each parameter set were checked for steady state by comparing corresponding data points from the last two APs; the cell was considered to be in steady state if the difference for each point in the AP was less than 5% of the maximum-minimum AP values. In almost all cases steady state was reached well before 1000 s; in those cases where steady state had not been reached, yet cell excitation was present, the simulation was continued to steady state (although it is worth noting that in cases where extended simulations were needed to reach steady state, the same would be true for cells in experiments).

Model output was measured using several commonly used biomarkers for describing AP morphology: maximum rate of membrane potential (V_m_) increase (dV_m_/dt_max_); diastolic V_m_ (V_rest_); V_m_ during the AP plateau (V_plat_), determined as the point after the spike in V_m_ due to activation at which dV_m_/dt_max_ reaches the smallest absolute value less than or equal to zero; and APD at 50% and 90% repolarisation (APD_50_ and APD_90_, respectively), measured as the time interval between the point of dV_m_/dt_max_ and the point when V_m_ was repolarised by 50% or 90% (*i.e.*, when V_m_ was less than or equal to V_rest_+0.5 or 0.1*[maximum V_m_−V_rest_]). In addition, the following biomarkers for describing changes in [Ca^2+^]_i_ were used: diastolic and systolic [Ca^2+^]_i_ ([Ca^2+^]_i_^dia^ and [Ca^2+^]_i_^sys^, respectively); amplitude of the Ca^2+^ transient (CaT), measured as [Ca^2+^]_i_^sys^-[Ca^2+^]_i_^dia^; and Ca^2+^ transient duration at 50% and 90% restoration of [Ca^2+^]_i_^dia^ (CTD_50_ and CTD_90_, respectively), measured as the time interval between the point of maximum rate of [Ca^2+^]_i_ increase and the point when [Ca^2+^]_i_ was reduced by 50% or 90% (*i.e.*, when [Ca^2+^]_i_ was less than or equal to [Ca^2+^]_i_^dia^+0.5 or 0.1*CaT). All analyses were performed using MATLAB (R2011b, version 7.13.0.564; Mathworks, Natick, MA).

### Constraining APD to Define a Physiological Population of Ventricular Models

In order to constrain the populations of models to those representing physiological variability, only parameter sets that produced APD_50_ and APD_90_ values that fell within the normal range for rabbit epicardium were included (these two biomarkers were chosen based on the availability of published experimental values). APD_90_ for rabbit epicardium has been well documented and a physiological range was readily established for all CLs [46]–[58]. Reports of APD_50_ values sufficient to derive a normal range, however, were not available at all CLs. Alternatively, values from the literature were used to establish a mean value for APD_50_ [48], [51] and the percentage change was assumed to be the same as for APD_90_. The resulting values are shown in Table 1.

**Table 1:** Normal range of rabbit epicardial APD_50_ and APD_90_ used to define physiological parameter sets.

	CL (ms)		
Biomarker	400	600	1,000
**APD_50_ (ms)**	104–135	116–159	137–188
**APD_90_ (ms)**	142–185	160–220	167–230

Values are derived from previously reported studies [46]–[58], as described in the text.

### Assessing the Accuracy of Biomarker Combinations for Determining the Goodness-of-fit of Generated Output to the Original Model

In order to investigate the relative importance of individual parameters to model response, as well as their interaction (described further in the next section), a method was established for comparing generated output to that of the original model. AP and Ca^2+^ transient morphology for each parameter set were directly compared to the original model by calculating the normalised root-mean-square deviation (NRMSD) between signals, defined as:
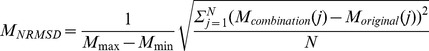
where *M*_max_ and *M*_min_ are the maximum and minimum V_m_ or [Ca^2+^]_i_ values for the original model, *M*_combination_(j) and *M*_original_(j) are the data points for V_m_ or [Ca^2+^]_i_ for a given parameter set and the original model, and *N* is the number of data points. Importantly, the normalisation step in this equation allowed V_NRMSD_ and Ca^2+^_NRMSD_ to be directly compared. By using data from the entire AP or Ca^2+^ transient, this method is a robust measure of goodness-of-fit; however, it is computationally expensive and poorly suited for comparison of model output with noisy experimental data. To address these limitations, the use of combinations of the calculated biomarkers was investigated. For each combination of biomarkers, the ∼250 parameter sets whose output most closely matched the output of the original model were determined by increasing the acceptable percentage difference until as close to 250 parameter sets were selected. Likewise, ∼250 parameter sets were selected based on the minimum combined V_NRMSD_ and Ca^2+^_NRMSD_. To quantify the ability of each biomarker combination to assess goodness-of-fit, the percentage overlap between the parameter sets selected by the two methods was calculated.

### Investigating the Interaction of Current Conductances and their Relative Importance to the Ventricular AP

The effect of simultaneously varying the magnitude of six current conductances can be thought of as comprehensively exploring a six-dimensional parameter space. In order to represent the resulting data as completely as possible, we employed a technique developed for studies of variability in the electrophysiology of neurons known as ‘clutter-based dimension reordering’ [35]–[38], that enables visualisation of higher dimensional parameter spaces in two dimensions. This method can be thought of as a linear projection of a multi-dimensional space to a lower dimensional space, with each point in *n*-dimensions assigned to a unique point in two-dimensions (much like slicing a cube and placing the resulting squares next to each other, only with ‘slices’ taken in more than three-dimensions, such that with continuous slicing the dimensionality of the space is iteratively reduced until it can be visualised in two-dimensions).

**Figure 1:**
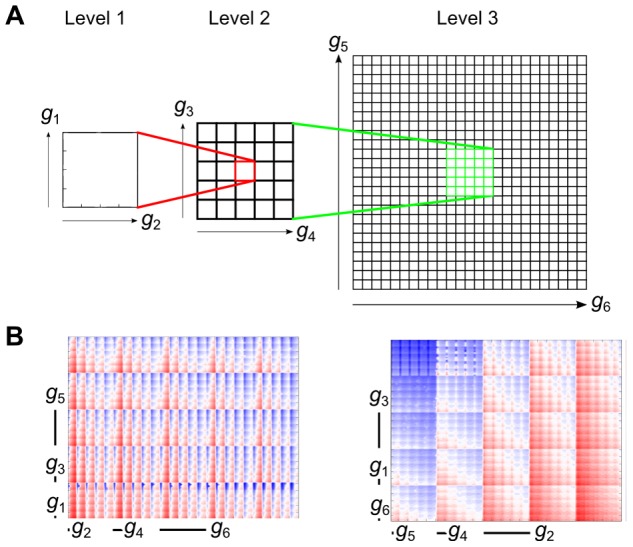
The dimensional stacking process. (A) The effect of two ‘low order’ conductances (*g*_1_ and *g*_2_) are plotted in a contour plot, with all other conductances held constant at their control value. This plot is then embedded in a larger grid spanning two ‘medium order’ conductances (*g*_3_ and *g*_4_). For each value of *g*_3,4_, the *g*_1,2_ plot is repeated for the respective values. This process is repeated to represent the two ‘high order’ conductances (*g*_5_ and *g*_6_). (B) Example showing a random stack order *(left)*, versus an optimised stack order *(right)* for the same variable.

The first step in clutter-based dimension reordering is ‘dimensional stacking’ (Fig. 1A). In our case, two of the conductances being varied were randomly chosen (*g*_1_ and *g*_2_), and with all other conductances held constant at control values, their effect on the measured biomarkers was displayed by a contour plot (‘Level 1’ in Fig. 1). Two other conductances were chosen (*g*_3_ and *g*_4_) and the original contour plot was repeated for each combination of these parameters. The subsequent plots were arranged in a grid reflecting the variation of *g*_3_ and *g*_4_ (‘Level 2’ in Fig. 1), *i.e.* the Level 1 plot that has *g*_3_ and *g*_4_ at their minimum values is at the bottom left of the Level 2 grid, and the Level 1 plot that has *g*_3_ and *g*_4_ at their maximum values is at the top right of the Level 2 grid. This process was then repeated for the last two conductances.

The resulting ‘dimensional stack’ was then optimised by rearranging the levels of the conductances (Fig. 1B). This was achieved by minimising the absolute difference between each point and its four neighbours in the *x* and *y* plane. The result of this optimisation was to ‘smooth’ the resulting plot, which leads to the ‘low order’ conductances (that have the smallest effect) being in Level 1, and the ‘high order’ conductances (that have the largest effect) being in Level 3. By determining the optimum ‘stack order’, patterns within the data are revealed. For instance, the greatest changes in the biomarker being considered are observed on the largest scale of the highest order conductances. It should be noted that in some cases the stack order before optimisation was used, to allow direct comparison of individual stacks for revealing inter-stack differences.

## Results

### Physiological Ventricular AP Variability can be Reproduced using a Population of Cell Models with Diverse Repolarising Current Conductances

By using the values derived from the literature to describe physiological ranges for APD_50_ and APD_90_ (Table 1), it was possible to constrain the combinations of current conductances to those producing experimentally measured variability at each CL. The numbers of parameter sets that produced a physiological output are given in Table 2, with the associated minimum, mean, and maximum values for all computed biomarkers presented in Table 3.

**Table 2:** Number of parameter sets producing both APD_50_ and APD_90_ values within the physiological range.

	CL (ms)						
Model	400	600	1,000	400∩600	400∩1,000	600∩1,000	400∩600∩1,000
**Mahajan**	3,946	1,031	0	577	0	0	0
**Shannon**	1,691	2,631	5,352	1,384	1,511	2,526	1,352
**Expanded Mahajan**	9,447	11,229	5,650	6,797	779	4331	779

Parameter values were varied by ±30% from the original parameter set, and then further for the Mahajan model (‘Expanded Mahajan’) as explained in the text. *x∩y* and *x∩y∩z* represent parameter sets that produce physiological values at a CL of *x* and *y*, or a CL of *x*, *y*, and *z*, respectively.

**Table 3:** Minimum, mean, and maximum values of all computed biomarkers produced with the physiological parameter sets.

Biomarker	CL (ms)	Model	Minimum	Mean	Maximum
**dV/dt_max_ (V/s)**	**400**	**Shannon**	233	302	329
		**Mahajan**	200	230	252
	**1000**	**Shannon**	278	327	352
		**Mahajan**	248	279	311
**V_rest_ (mV)**	**400**	**Shannon**	−92	−88	−82
		**Mahajan**	−88	−86	−85
	**1000**	**Shannon**	−88	−86	−81
		**Mahajan**	−89	−88	−87
**APD_50_ (ms)**	**400**	**Shannon**	112	126	135
		**Mahajan**	104	108	117
	**1000**	**Shannon**	137	155	181
		**Mahajan**	165	182	188
**APD_90_ (ms)**	**400**	**Shannon**	143	154	169
		**Mahajan**	142	155	185
	**1000**	**Shannon**	167	186	217
		**Mahajan**	207	223	230
**[Ca^2+^]_i_^dia^ (µM)**	**400**	**Shannon**	1.24	1.29	1.32
		**Mahajan**	0.29	0.37	0.47
	**1000**	**Shannon**	0.82	0.84	0.86
		**Mahajan**	0.15	0.16	0.18
**[Ca^2+^]_i_^sys^ (µM)**	**400**	**Shannon**	4.49	4.92	5.22
		**Mahajan**	1.43	2.78	4.46
	**1000**	**Shannon**	3.13	3.33	3.68
		**Mahajan**	0.41	0.59	0.76
**CaT (µM)**	**400**	**Shannon**	3.25	3.63	3.91
		**Mahajan**	1.14	2.41	4.00
	**1000**	**Shannon**	2.30	2.49	2.82
		**Mahajan**	0.26	0.42	0.58
**CTD_50_ (ms)**	**400**	**Shannon**	123	130	134
		**Mahajan**	133	139	147
	**1000**	**Shannon**	139	150	157
		**Mahajan**	208	231	268
**CTD_90_ (ms)**	**400**	**Shannon**	263	267	271
		**Mahajan**	245	249	260
	**1000**	**Shannon**	382	394	404
		**Mahajan**	460	510	585

With the Shannon model, there existed at least one parameter set that produced a physiological output at each CL, with some of these generating a physiological output at all CLs (interestingly, however, this did not include the original parameter set, demonstrating that the original Shannon model does not reproduce physiological output under some conditions). On the other hand, while a relatively large number of parameter sets produced a physiological output at a CL of 400 ms with the Mahajan model (the CL for which the Mahajan model was designed), fewer parameter sets matched at a CL of 600 ms, and *none* at a CL of 1,000 ms (the increase in APD with increasing CL was disproportionately large).

In order to address the failure of the Mahajan model in finding parameter sets that generated a physiological output with increased CL, the range of conductance variation was expanded. To determine an appropriate new parameter space to be explored, first the ranges of APD_50_ and APD_90_ used for constraint were moderately extended (±10%) and the model population was compared to this new range. This extended range of APD resulted in 24 matches with outputs at a CL of 1,000 ms. Based on the distribution of the conductance values that were present in these matching parameter sets, the range of conductance values for each current was expanded by following the underlying trend. The expanded ranges chosen were: *g*_to_ = −15%–+45%; *g*_Ca,L_ = +30%–+90%; *g*_Kr_ = −15%–+45%; *g*_Ks_ = +45%–+105%; *g*_K1 = _−15%–+45%; *g*_NaK_ = −45%–+15%. A new parameter search was then performed as before, using the original physiological ranges for APD and with current conductances varied in 15% increments across their new range, resulting in an additional 15,625 parameter sets at each CL. In the case of this expanded search, the number of parameter sets that matched at *all* CLs was approximately half of that with the Shannon model.

**Figure 2:**
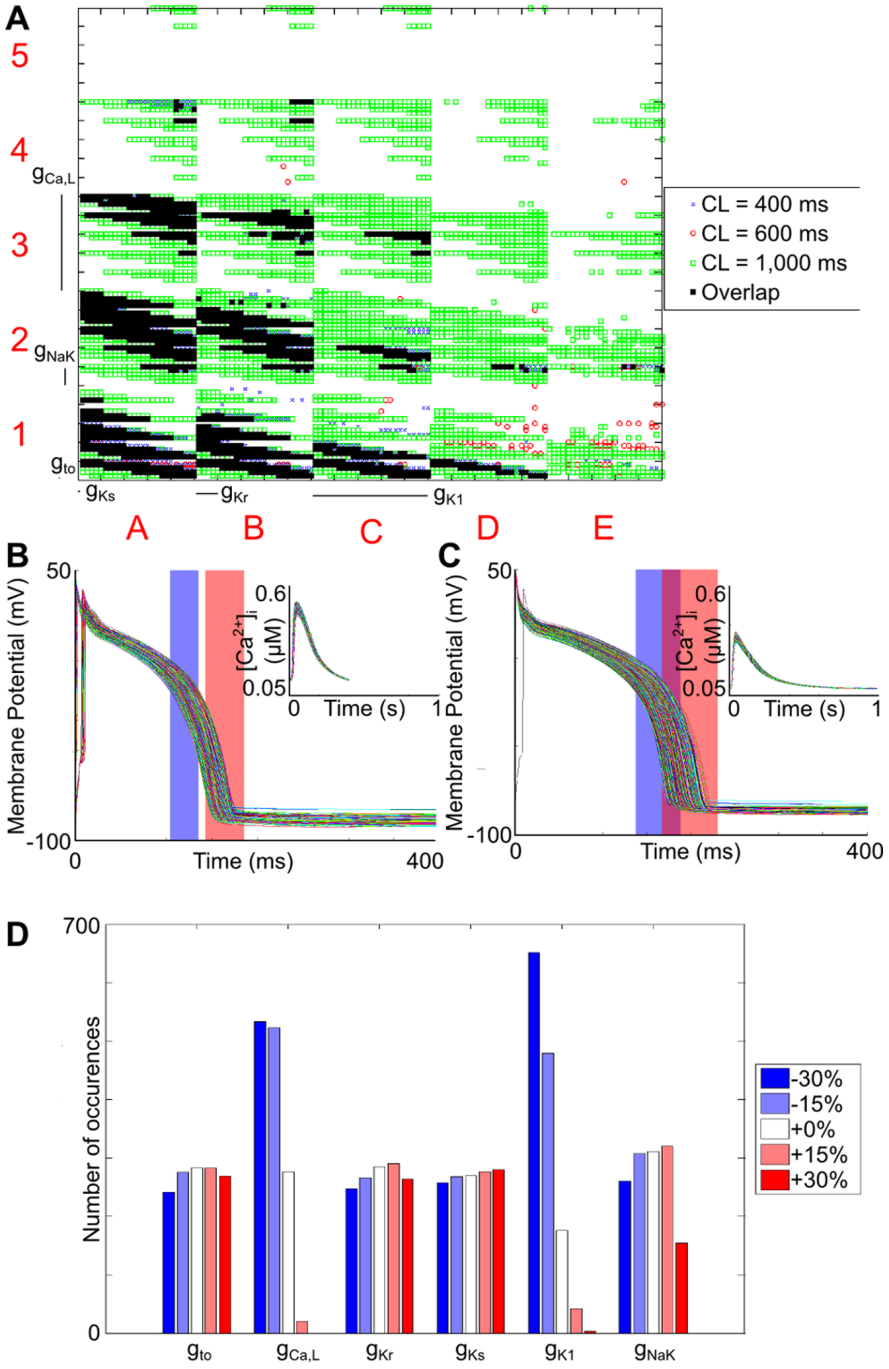
Parameter sets for the Shannon model that produce values of both APD_50_ and APD_90_ that fall within the experimentally derived range. (A) Dimensional stack image showing the location of matching parameter sets for each CL and their overlap. (B) V_m_ and [Ca^2+^]_i_ (inset) profiles for the matching parameter sets at a CL of 400 ms. (C) V_m_ and [Ca^2+^]_i_ (inset) profiles for the matching parameter sets at a CL of 1,000 ms. (D) Distribution of conductance values for the valid parameter sets. For both (B) and (C), the physiological ranges of APD_50_ and APD_90_ are represented by the blue and red rectangles, respectively.

The parameter sets producing a physiological output with the Shannon model are shown using a dimensional stack in Fig. 2, along with the generated V_m_ and [Ca^2+^]_i_ profiles at a CL of 400 and 1,000 ms, and the associated distribution of conductance values. The most obvious trend was that parameter sets producing a physiological output generally had a simultaneous reduction in both *g*_Ca,L_ and *g*_K1_. This is evident in both Fig. 2A, demonstrated by a clustering of the valid parameter sets at the bottom left of the dimensional stack, and from the associated distribution of conductances, shown in Fig. 2D. It also appears that the distribution of the other conductances was fairly even, however a closer examination of the dimensional stack in Fig. 2A reveals trends between the parameters (demonstrating the power of the clutter-based dimension reordering technique for visualisation of multi-dimensional parameter spaces). For instance, within the *g*_NaK_/*g*_Kr_ surfaces (Level 2 of the stack), the matching parameter sets are spread in an approximately diagonal line from top left to bottom right, indicating that when *g*_NaK_ was increased, this was offset by a decrease in *g*_Kr_, and *vice versa*. As *g*_Ca,L_ and *g*_K1_ decrease, this diagonal line moves further towards the bottom left corner, indicating that a further reduction of *g*_NaK_ and *g*_Kr_ was required to continue to produce a physiological output.

The effect of *g*_to_ was more complicated. When *g*_Ca,L_ and *g*_K1_ were reduced by 30%, and *g*_NaK_ was also reduced, matching parameter sets then included those with an increased *g*_to_. The opposite was true when *g*_NaK_ was increased, as in these cases a decrease in *g*_to_ was necessary (square A1 in Fig. 2A). As *g*_K1_ was increased, fewer parameter sets with an increased *g*_NaK_ were valid, such that an increase in *g*_to_ was observed (squares B1, C1, and D1 in Fig. 2A). However, in all cases where *g*_Ca,L_ was not reduced by 30%, the opposite was true: parameter sets including reduced *g*_NaK_ and increased *g*_to_ were no longer valid (squares A2 and A3 in Fig. 2A). Finally, in all valid parameter sets as *g*_Kr_ was increased, *g*_to_ decreased. On the other hand, there appeared to be no limitations on the values of *g*_Ks_.

**Figure 3:**
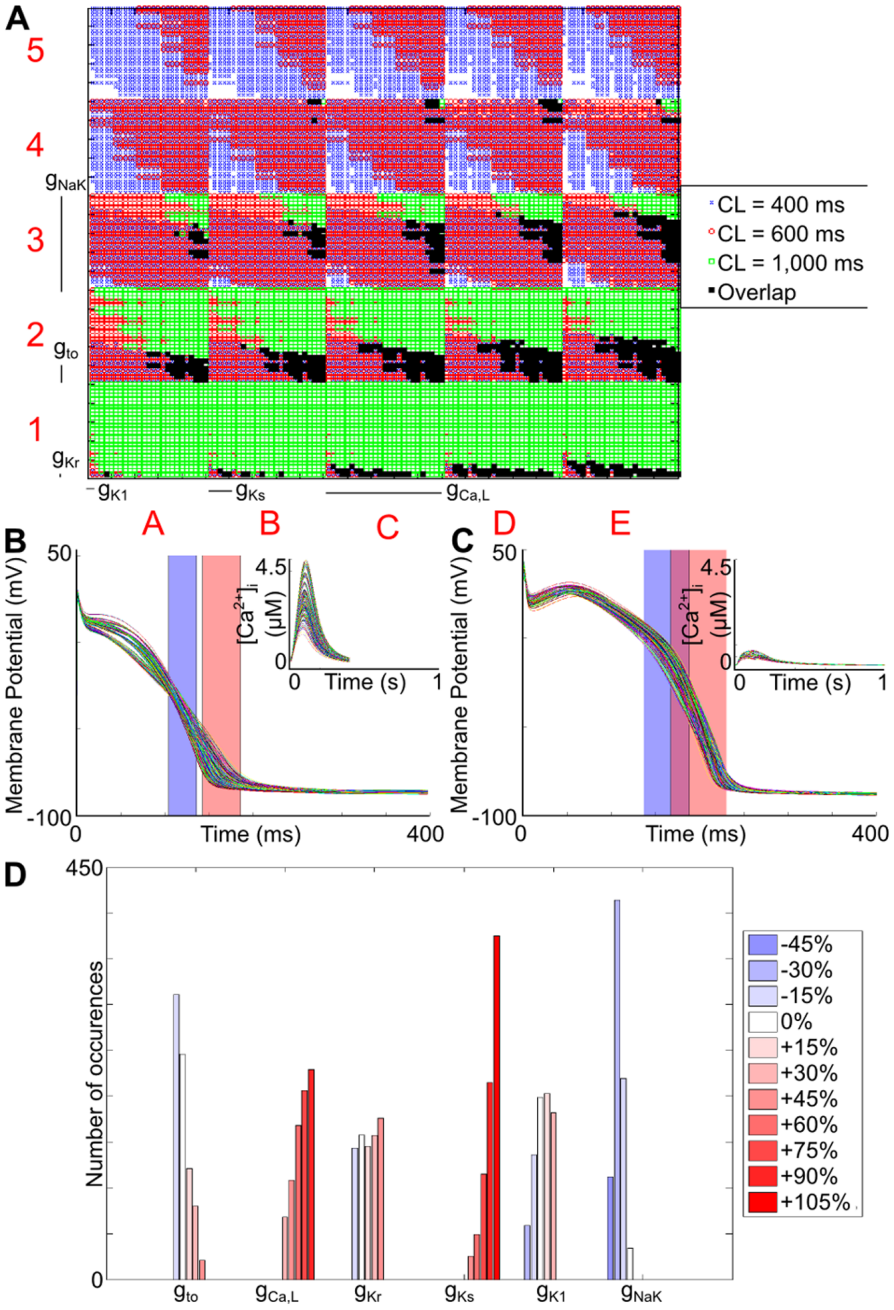
Parameter sets for the expanded Mahajan search that produce values of both APD_50_ and APD_90_ that fall within the experimentally derived range. (A) Dimensional stack image showing the location of matching parameter sets for each CL and their overlap. (B) V_m_ and [Ca^2+^]_i_ (inset) profiles for the matching parameter sets at a CL of 400 ms. (C) V_m_ and [Ca^2+^]_i_ (inset) profiles for the matching parameter sets at a CL of 1,000 ms. (D) Distribution of conductance values for the valid parameter sets. For both (B) and (C), the physiological ranges of APD_50_ and APD_90_ are represented by the blue and red rectangles, respectively.

For the expanded Mahajan search, the parameter sets producing a physiological output, the generated cellular profiles, and the distribution of valid conductance values are shown in Fig. 3. There are some differences compared to the Shannon model. For instance, with the Shannon model, *g*_Ks_ appeared to have no effect in determining the validity of parameter sets, while with the Mahajan model it had a strong influence, as most matching parameter sets included the largest conductance variation (+105%). The opposite was true for *g*_K1_: while it had a large influence with the Shannon model, it was relatively unimportant with the Mahajan model. Similarly, with the Shannon model, *g*_to_ and *g*_NaK_ generally varied in the opposite direction, while with the Mahajan model they changed in the same direction.

With the Mahajan model there was also a strong correlation between *g*_NaK_ and *g*_Ks_, such that when *g*_NaK_ was increased, *g*_Ks_ also increased (demonstrated by a shift of matching parameter sets from the predominantly lower left corner to the upper right corner of level two plots; for instance, compare the distribution within E1 and E3). On the other hand, there appeared to be no limitations on the values of *g*_Kr_.

### A Combination of APD_50_, APD_90_, and CaT Provides the Most Accurate Measure of Goodness-of-Fit

**Figure 4:**
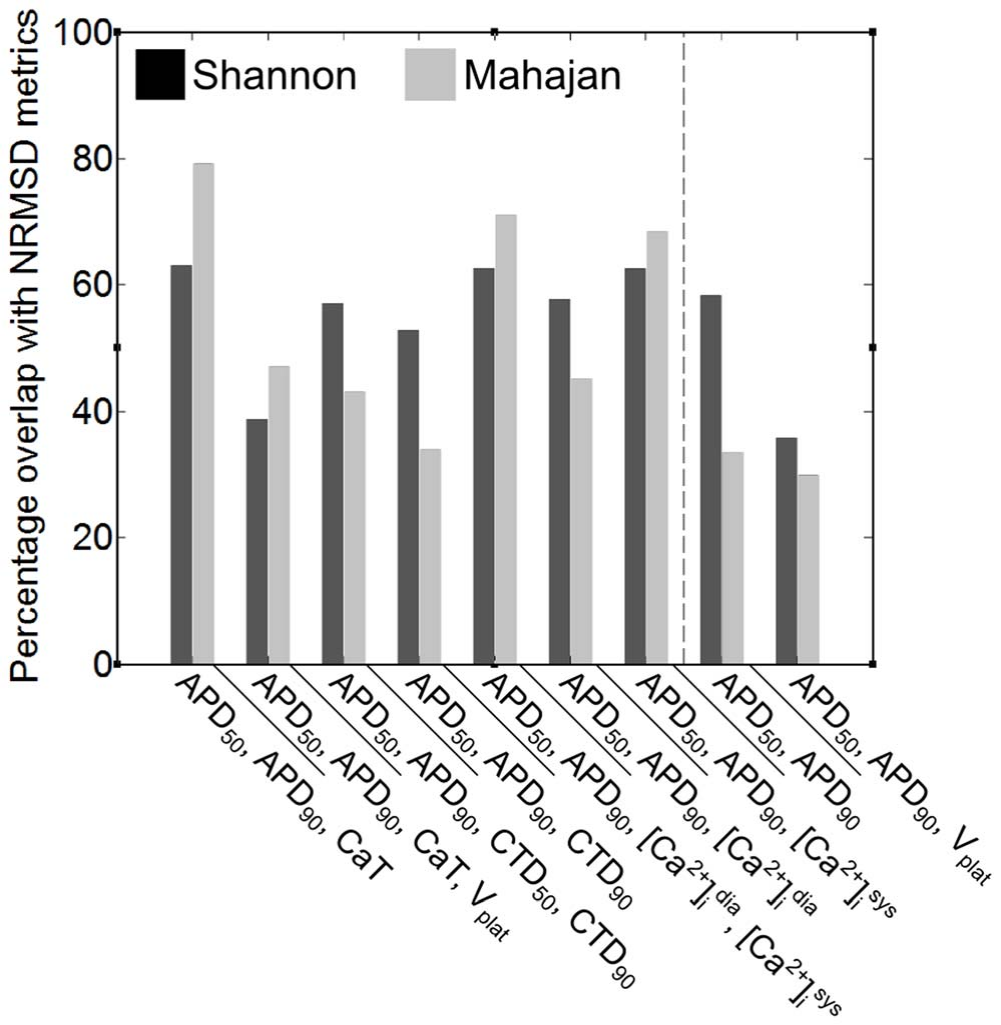
Percentage overlap of matches between original model output and model output generated using the ∼250 parameter sets determined by the NRMSD metrics ([V_NRMSD_+Ca^2+^_NRMSD_]) and by combinations of biomarkers. While all combinations of biomarkers were tested, those shown represent the combinations with a relatively high percentage overlap. Combinations to the left of the dashed line include information about both V_m_ and [Ca^2+^]_i_, while those to the right include only V_m_ data.

In order to investigate the relative importance of individual parameters and their interaction (reported in the next section), a method for comparing generated APs to the original model using combinations of biomarkers was defined. Fig. 4 shows the percentage overlap of parameter sets whose output was determined to match the original model output by the NRMSD metrics and by combinations of biomarkers. It can be seen that when [Ca^2+^]_i_-based biomarkers were included, there was greater overlap. The percentage overlap for the Shannon and Mahajan models was not consistent. Overall, however, a combination of APD_50_, APD_90_, and CaT was most accurate (with a combination of APD_50_ and APD_90_ also showing a relatively high percentage overlap). Consequently, a combination of APD_50_, APD_90_, and CaT was used to assess goodness-of-fit.

### Relative Importance of Current Conductances and their Interaction is Rate-Dependent

**Figure 5:**
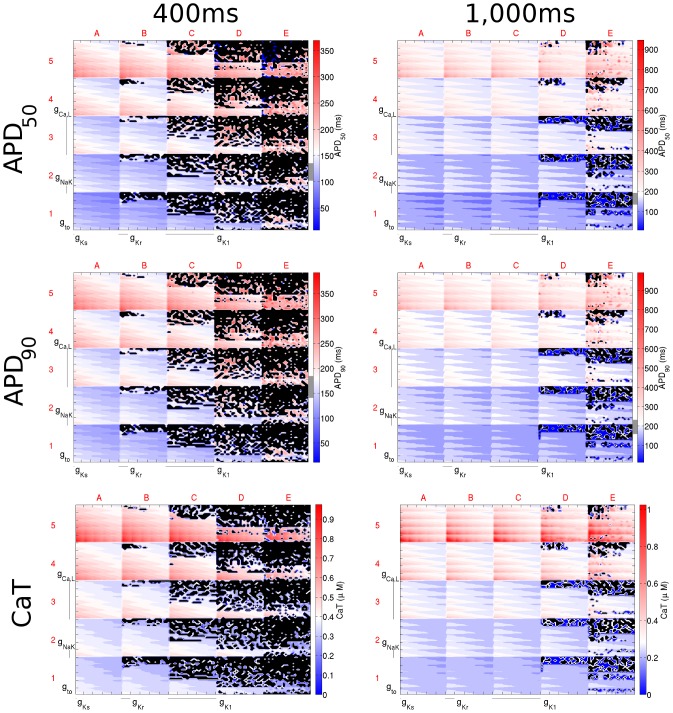
Dimensional stack images demonstrating the effect of simultaneously varying the magnitude of six repolarising current conductances in the Shannon model. The top, middle, and bottom rows show the effects on APD_50_, APD_90_, and CaT, respectively. The left column is based on simulations with a CL of 400 ms and the right with a CL of 1,000 ms. In the contour plots, red represents an increase from the control value, blue a decrease, and white no change. The physiological range of APDs determined from the literature is represented by the grey region next to the colour bars in each panel. Black dots represent parameter sets with which the model did not reach steady state. In this case the optimum stack orders are not displayed; instead the order before optimisation has been used, which allowed direct comparison of the stacks to reveal differences in effects on each biomarker.

**Figure 6:**
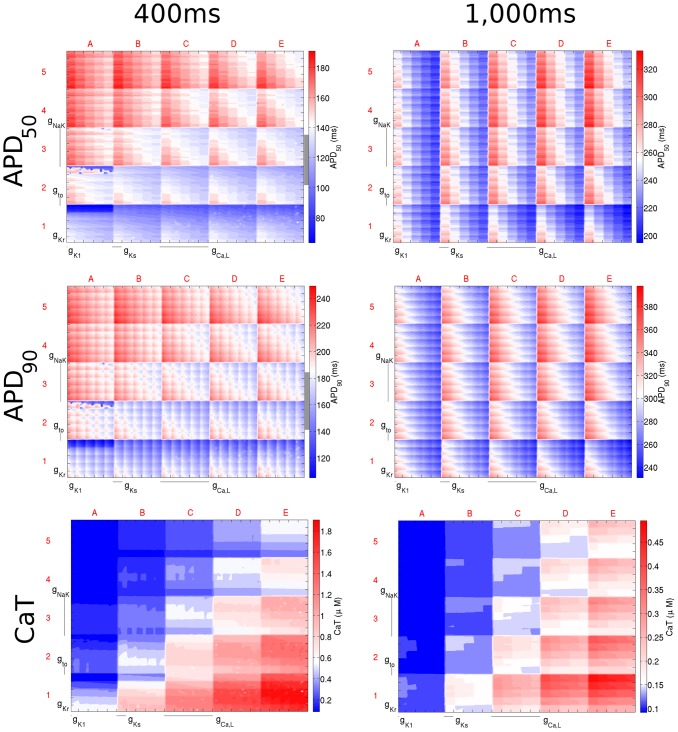
Dimensional stack images demonstrating the effect of simultaneously varying the magnitude of six repolarising current conductances in the Mahajan model. The top, middle, and bottom rows show the effects on APD_50_, APD_90_, and CaT, respectively. The left column is based on simulations with a CL of 400 ms and the right with a CL of 1,000 ms. In the contour plots, red represents an increase from the control value, blue a decrease, and white no change. The physiological range of APDs determined from the literature for a CL of 400 ms is represented by the grey region next to the colour bars in each panel (the grey region is absent for a CL of 1,000 ms as the APD values fell outside of the physiological range). In this case the optimum stack orders are not displayed; instead the order before optimisation has been used, which allows direct comparison of the stacks to reveal differences in effects on each biomarker.

As demonstrated by the differences in the dimensional stacks presented in Figs. 5 and 6 (summarised as differences in optimum stack order in Table 4), the relative importance of the varied current conductances was dependent on the CL. In considering this, it should be noted that the non-linear interaction between currents and the subsequent changes in ion concentrations and AP waveform, often resulted in different changes in current magnitudes than might be expected from the change in current conductance. For instance, when *g*_Ks_ was subjected to ±30% variation at a CL of 1,000 ms, the amplitude of the slow delayed rectifier K^+^ current varied from −99% to +386%.

**Table 4:** Optimum stack order for APD_50_, APD_90_, and CaT with the two rabbit-specific ventricular AP models.

			Optimum Stack Order (*x*, *y*)		
Model	Biomarker	CL (ms)	Low Order→High Order		
**Shannon**	**APD_50_**	**400**	(*g*_to_, *g*_Ks_)	(*g*_NaK_, *g*_Kr_)	(*g*_Ca,L_, *g*_K1_)
		**1,000**	(*g*_K1_, *g*_Ks_)	(*g*_NaK_, *g*_Kr_)	(*g*_Ca,L_, *g*_to_)
	**APD_90_**	**400**	(*g*_to_, *g*_Ks_)	(*g*_NaK_, *g*_Kr_)	(*g*_Ca,L_, *g*_K1_)
		**1,000**	(*g*_K1_, *g*_Ks_)	(*g*_NaK_, *g*_Kr_)	(*g*_Ca,L_, *g*_to_)
	**CaT**	**400**	(*g*_K1_, *g*_Ks_)	(*g*_NaK_, *g*_to_)	(*g*_Ca,L_, *g*_Kr_)
		**1,000**	(*g*_K1_, *g*_Ks_)	(*g*_NaK_, *g*_Kr_)	(*g*_Ca,L_, *g*_to_)
**Mahajan**	**APD_50_**	**400**	(*g*_Kr_, *g*_K1_)	(*g*_Ks_, *g*_Ca,L_)	(*g*_to_, *g*_NaK_)
		**1,000**	(*g*_Kr_, *g*_K1_)	(*g*_to_, *g*_Ca,L_)	(*g*_Ks_, *g*_NaK_)
	**APD_90_**	**400**	(*g*_Kr_, *g*_Ca,L_)	(*g*_K1_, *g*_Ks_)	(*g*_to_, *g*_NaK_)
		**1,000**	(*g*_Kr_, *g*_K1_)	(*g*_Ks_, *g*_to_)	(*g*_Ca,L_, *g*_NaK_)
	**CaT**	**400**	(*g*_Kr_, *g*_K1_)	(*g*_to_, *g*_Ks_)	(*g*_NaK_, *g*_Ca,L_)
		**1,000**	(*g*_Kr_, *g*_K1_)	(*g*_Ks_, *g*_to_)	(*g*_NaK_, *g*_Ca,L_)

Each pair of parameters represents low, medium, or high order current conductances. For each pair, the first component is plotted on the *x*-axis and the second component on the *y*-axis. The (*x*,*y*) order can be reversed without affecting the result only if *all* (*x*,*y)* pairs are reversed.

For the Shannon model, the relative importance of *g*_to_ increased, and that of *g*_K1_ decreased, with CL (demonstrated by the optimum stack orders presented in Table 4). On the other hand, at most CLs, *g*_Ca,L_ and *g*_Ks_ were the highest and lowest order conductances and *g*_Kr_ and *g*_NaK_ were generally of medium order.

For the Mahajan model, as CL increased, the relative importance of *g*_to_ decreased. This was opposite to the response seen with the Shannon model. At the same time, *g*_Ks_ became more influential, despite little change in its position in the optimum stack order. This can be seen by examining the difference between the dimensional stacks with CLs of 400 and 1000 ms shown in Fig. 6. At a CL of 400 ms, the greatest effect on APD (represented by deep red and blue) is seen at the edges of the dimensional stack image (squares A1, A5, E1, and E5), indicating an extreme increase/decrease in *g*_Ca,L_ and *g*_NaK_. However, with an increase in CL, the maximum effect of the change is seen throughout the dimensional stack, as a result of the increased importance of *g*_Ks_. In contrast, the relative importance of *g*_NaK_ and *g*_Kr_ were independent of CL, being consistently one of the highest and lowest order conductances.

### Biomarker Variability is Rate-Dependent

**Figure 7:**
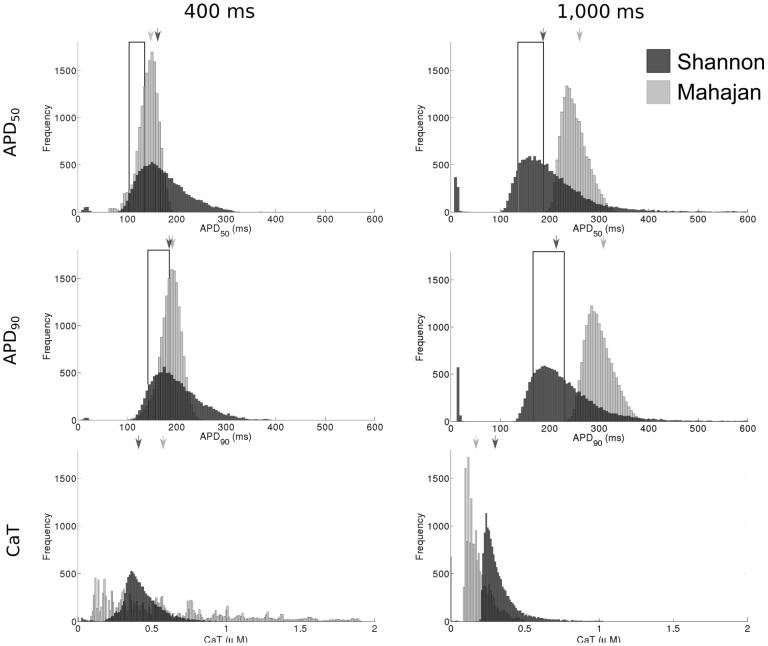
Histograms showing the range of APD_50_, APD_90_, and CaT in the model populations. The value generated with the control parameter set for each model is indicated by the arrow. The physiological range of APD_50_ and APD_90_ derived from the literature are represented by the boxed area.

Histograms showing the variability of APD_50_, APD_90_, and CaT across all combinations of current conductances are shown in Fig. 7. The Mahajan and Shannon models demonstrated similar distributions for both APD_50_ and APD_90_ (upper and middle panels in Fig. 7). They differed, however, in that the Mahajan model demonstrated more narrow distributions than the Shannon model, while the Shannon model generated more APD values that fell within the physiological range (discussed in the first section above). For the Shannon model, the shape of the APD_50_ and APD_90_ distributions were relatively well conserved between a CL of 400 and 1,000 ms, other than an increase in the number of matching parameter sets. In contrast, the Mahajan model demonstrated a widening of the APD_50_ and APD_90_ distributions, as well as an increase in their mean. In the case of simulations with a CL of 1,000 ms, the increase in mean APD was such that the entire distribution fell outside of the physiological range.

The change in CaT distribution with a change in CL was more dramatic (lower panels in Fig. 7). For the Shannon model, the distribution narrowed with an increase in CL. The distribution with the Mahajan model followed a similar pattern, however with an even larger change. At a CL of 400 ms, the range of CaT was very broad (∼0.1 µM to ∼1.9 µM), indicating that CaT was relatively poorly constrained within the parameter space. When CL was increased, however, the range was greatly reduced (∼0.1 µM to ∼0.5 µM).

## Discussion

This study examined the effects of variability in six transmembrane current conductances on rabbit ventricular AP repolarisation (represented by APD). This was accomplished by performing a systematic exploration of the multi-dimensional parameter space using distributed computing software with two biophysically-detailed computational models of the rabbit ventricular AP. The results were used to determine a population of models that reproduced experimentally reported intercellular variability in APD at a variety of CLs. It was shown that the populations of models were able to produce physiological ventricular APs with a wide range of conductance values. Specialised techniques for visualisation of the multi-dimensional spaces revealed interaction of individual current conductances, as well as their relative importance for APD. It was demonstrated that this interaction and influence, as well as biomarker variability, were rate- and model-dependent. Specifically, with the Shannon model, *g*_Ca,L_ had the greatest influence on APD variability at both 400 and 1000 ms, along with *g*_K1_ and *g*_to_ at 400 and 1000 ms, respectively. With the Mahajan model, on the other hand, *g*_NaK_ had the greatest influence on APD variability at both 400 and 1000 ms, along with *g*_to_ and *g*_Ca,L_ at 400 and 1000 ms, respectively.

Understanding the effects of individual current conductances was facilitated by the use of clutter-based dimension reordering [35]–[38]. For instance, this technique revealed the importance of *g*_to_ to AP biomarkers, as well as its interactions with the other conductances. These effects could not have been experimentally elucidated, and thus are not evident without the use of this technique. Further, as shown in Figs. 2 and 3, the combinations of conductances that produced physiological outputs were not equally distributed across all conductances. This reflects both a difference in their relative importance for the ventricular AP, as well as non-linear, interactions in the currents that they affect. However, due to the complex relationship between the various conductances tested, trends were not generally evident from distributions alone.

The results presented in Figs. 5 and 6, and summarised in Table 4, demonstrate the influence of CL on the relative importance of various current conductances on the ventricular AP, along with their interactions. As described above, the optimum stack order, which is an indication of the relative importance of the individual conductances to the AP and illustrates their interrelation, changed with CL. Changes, however, were unpredictable. The most extreme example of an unexpected change in the relative importance of a conductance to the AP was the change seen for the influence of *g*_to_ on APD_50_ and APD_90_ with the Shannon model. At a CL of 400 ms, *g*_to_ was a low-order conductance (reflecting a low importance), while at a CL of 1,000 ms, it became a high-order conductance. The importance of some conductances, on the other hand, changed little with CL. For instance, *g*_Ca,L_ was consistently of high-order and *g*_Ks_ of low-order. The importance of CL in determining the effects of current conductance variability on AP biomarkers highlights the need to consider rate in experimental investigations, as well as for adequately constraining model populations for computational studies of variability.

Previous efforts to relate variability in current conductances to ventricular AP variability have focused on three approaches: i) the inclusion of stochasticity in model formulations; ii) the use of parameter sensitivity analyses; and iii) the generation of model populations (as in the present paper). Regarding the use of stochastic formulations, studies by Pueyo *et al.* [3] and Sato *et al.* [8] have investigated the effect of stochastic gating of the slow delayed rectifier K^+^ current on human, guinea pig, and rabbit ventricular AP repolarisation, and studies by Tanskanen *et al.* [9] and Hashambhoy *et al.* [5] have done the same for stochastic gating of the L-type Ca^2+^ current and sarcoplasmic-reticulum Ca^2+^ release in a canine model; these, however, considered variability in no other currents. Lemay *et al.*, on the other hand, did include stochastic gating in multiple currents of a guinea pig model, but this was a purely computational investigation, without the use of experimental data for model constraint [6]. In a study by Walmsley *et al.* stochasticity was included in the gating variables of a phenomenological guinea pig ventricular AP model, though this did not allow for consideration of the effects of specific currents [4]. Examples of the use of parameter sensitivity analysis come from studies by Romero *et al.* [11], [12] that investigated the role of ionic current variability on biomarkers in human and rabbit models, yet these were limited as they focused on variation of only one current at a time, missing potential important current interactions. Work by Sarkar and Sobie. has employed a combination of sensitivity analysis and model populations with human and canine models, but this work was restricted to random sampling of a narrow parameter space close to the control model conditions [13], [14], [19]. A population of 19 canine models was utilised by Davies *et al.* to capture variability of the canine ventricular AP, however this represents only a small population [16]. Thus, there remains the need for investigations of the effects of simultaneous variation of current conductances on ventricular repolarisation over the entire physiological range.

This was recently addressed for the rabbit Purkinje AP by Britton *et al.* [15]. In this study, a methodology was developed to simulate the complete range of observed AP variability by randomly varying current conductances and channel kinetics across a wide range of values. By reducing the resulting population of over 10,000 models down to 213 using experimental data, it was shown that a wide range of parameter values could produce a physiological Purkinje AP. Interestingly, while the results from that study demonstrated non-uniform distributions of some current conductance across the population of models, as in the present work, they did not show any obvious relationships between currents by pair-wise correlation analysis. This is in contrast to the current study, in which important interactions between currents were revealed by projecting the multi-dimensional parameter space onto a two-dimensional representation using clutter-based dimension reordering [35]–[38]. This re-emphasises the power of this technique, as previously shown in neuroscience applications [59]–[62], for revealing unsuspected compensatory mechanisms that may contribute to normal cellular function. Differences in outcomes of the two studies may relate to other important methodological factors, such as effects of generating model populations by random (as in Britton *et al.*) *versus* systematic (as in the current study) sampling of the parameter space. The study by Britton *et al.* employed the Latin hypercube sampling method [63], which generates parameter sets without bias, but only provides a random subset of the entire parameter space, while the present work utilised the Nimrod/G distributed computing grid platform [32], [34] to perform a systematic exploration of the complete parameter space. Finally, it should be noted that dissimilarities may also represent differences between Purkinje and ventricular electrophysiology and their model representations.

While the results presented here suggest that variation in current conductances over a wide range of values may account for normal variability in rabbit ventricular AP repolarisation, other factors may be involved. One of the underlying assumptions of this study was that AP variability is primarily a result of differences in the relative magnitude of currents, rather than underlying current dynamics, which were not varied. Changes in channel properties other than conductance could result in similar changes in AP biomarkers and also account for some of the experimentally observed variability. A study by Romero *et al.*, in which a one-dimensional sensitivity analysis of the rabbit-specific models used in this study was performed with a similar range of parameter variation, showed that along with repolarisation currents, APD was significantly modified by changes in the activation and inactivation rates of the associated channels [11]. At the same time, further constraints to the model populations (for example matching of rate-adaption or restitution properties), as well as consideration of additional biomarkers (for instance relating to intracellular ion concentrations), may be necessary to ensure their applicability to additional physiological states. This has been recently demonstrated in a study by Walmsley *et al.*, in which populations of failing and non-failing human ventricular myocytes with variation in current conductances were compared using various biomarkers at numerous CLs to investigate which currents drive variability in the two cell populations [64]. Finally, as the range and resolution of parameter space sampling in the present study was limited by computational tractability, there may be additional influences and interactions of current conductances important for ventricular AP variability that were not appreciated. As mentioned above, it is not possible to assess the physiological ranges of the current conductances investigated in this study, so that they may be related to the values included in the calibrated populations of models. We initially varied all conductances by ±30%, yet it was necessary with the Mahajan model to expand this range to generate a physiological output. Other computational studies have used a larger range of conductances than in the present study ([14]–[16]), possibly representing the true physiological range of values, and supporting the expanded range used with the Mahajan model.

While providing insights into rabbit ventricular AP variability, the present study also represents an evaluation of the two most commonly used rabbit-specific ventricular AP models. As has been shown previously by Romero *et al.* [11], the effects and relative importance of current conductance variation in the present study were largely model-dependent. Also, while the models produced a physiological output with their original parameter set under the conditions for which they were designed, when CL was varied this was not the case. These rate and model-dependent differences highlight the importance of careful model selection, validation, and appropriate use for computational studies [65], [66].

In summary, we have demonstrated that experimentally observed intercellular variability of the rabbit ventricular AP can be reproduced by a population of computational models that includes large variations in current conductances important for repolarisation. Importantly, this work moved beyond previous investigations of ventricular repolarisation variability by a systematic exploration of the multi-dimensional conductance space with the use of distributed computing software and specialised visualisation techniques. This represents a robust method for investigating the interaction of current conductances and their relative importance to the ventricular AP, an important step towards a better understanding of intercellular variability in ventricular repolarisation. Importantly, this can only be achieved using a combination of advanced computational modelling and experimental calibration. In the future, similar model populations may be applied to tissue and organ level studies of diseased states to better understand the role of intercellular repolarisation variability in the generation of ventricular arrhythmias.
